# Analysis of risk factors and treatment strategies for lumbar cistern blockage after craniocerebral surgery

**DOI:** 10.3389/fnins.2023.1124395

**Published:** 2023-02-28

**Authors:** Min Zheng, Qilong Tian, Xuejiao Wang, Liqin Liu, Xiurui Deng, Yan Qu, Qing Cai

**Affiliations:** Department of Neurosurgery, Tangdu Hospital, Air Force Medical University, Xi’an, China

**Keywords:** lumbar cistern drainage (LCD), lumbar cistern blockage, risk factors, treatment strategies, surgery

## Abstract

**Objective:**

Lumbar cistern blockage is a common complication of continuous lumbar cistern drainage. This paper analyzes the risk factors for lumbar cistern blockage drainage due to various causes and proposes a series of prevention and intervention measures to reduce blockage or improve recanalization after blockage.

**Methods:**

The clinical data of 637 patients with various lesions who underwent lumbar cistern drainage in our hospital were retrospectively collected and analyzed. Perioperative clinical and imaging data were assessed. Variates were analyzed using univariate and multivariate logistic regression analyses.

**Results:**

A total of 13.7% (87/637) of patients had lumbar cistern blockage. Multivariate analysis revealed that drainage time (≥7 days), CSF volume <200 (mL/d), CSF leakage, and abnormal CSF properties were predictors of lumbar cistern blockage. Reducing the probability of lumbar cistern blockage can be achieved by repeatedly flushing, increasing the drainage flow and shortening the drainage time. The recanalization rate after blockage was 67.8% (59/87). After the drainage tube was removed, no complications related to the drainage tube occurred during the 1-week follow-up.

**Conclusion:**

Lumbar cistern blockage is the main reason for poor drainage. Prevention or early intervention can effectively reduce the probability of blockage and achieve the purpose of drainage of cerebrospinal fluid.

## Introduction

Lumbar cistern drainage (LCD) is used to treat intracranial infection, vasospasm after hemorrhage, intractable intracranial hypertension after craniocerebral trauma, cerebrospinal fluid leakage, and other related diseases (Wolf, 2015; Chen et al., 2017; Zwagerman et al., 2018; Badhiwala et al., 2021). It is widely used in the field of neurosurgery because of its reliable effect and obvious shortening of the course of disease. The premise for achieving therapeutic effects of lumbar cistern drainage is to maximize the drainage volume of cerebrospinal fluid and avoid complications such as headache and even cerebral herniation caused by excessive drainage volume (Sugrue et al., 2009).

The study confirmed that the drainage volume of cerebrospinal fluid (250–300 ml/d) is safe and effective (Ma et al., 2021). Insufficient drainage volume may affect the treatment effect and prolong the hospital stay. The most important reason for insufficient drainage is lumbar cistern blockage. After blockage, the cerebrospinal fluid cannot be drained smoothly or cannot flow out, which increases the treatment time. Replacing the lumbar cistern tube will increase cost for patients, and even more seriously, it may lead to intracranial infection and aggravate the condition of patients. Therefore, the goal is to prevent lumbar cistern blockage or recanalization after blockage to ensure continuous lumbar cistern drainage, and to avoid many complications after lumbar cistern drainage.

Although lumbar cistern drainage is a common and minimally invasive neurosurgery procedure, there is no systematic study on the risk factors for lumbar cistern blockage, and there are few reports on the methods of recanalization after blockage. This paper retrospectively analyzed 637 patients who required lumbar cisterna drainage after craniocerebral surgery for various reasons in our department, determined the risk factors for lumbar cisterna blockage and introduced the treatment experience of recanalization after blockage to provide guidance and reference values for clinical prediction.

## Materials and methods

### Patient selection

A retrospective study was performed on 637 patients who underwent lumbar drainage for various reasons in the Department of Neurosurgery, Tangdu Hospital of the Air Force Medical University (Xi’an, China) from July 2012 to November 2022. We collected patient data from radiology systems and electronic medical records. The criteria for inclusion were as follows: (1) Fisher grade 3 and 4 subarachnoid hemorrhage; (2) partial ventricular hemorrhage; (3) antimicrobial therapy for central nervous system infections; and (4) adjuvant treatment of cerebrospinal fluid leakage. Exclusion criteria were as follows: (1) severe increase in intracranial pressure; (2) puncture site lumbar deformity or bone destruction, resulting in lumbar puncture or catheterization difficulty; (3) dying individuals with severe systemic infections (e.g., severe sepsis), shock or on the verge of shock, and unstable vital signs; (4) cerebrospinal fluid circulatory pathway incomplete obstruction; and (5) patients with restless or abnormal mental behavior who cannot cooperate with the diagnosis and treatment. All the study procedures were approved by the ethics committee of Tangdu Hospital and followed the guidelines of the Helsinki Declaration.

### Variables and data collection

All patient data were collected from the hospital electronic medical records. Follow-up data were obtained *via* telephone interviews. Clinical data, such as age, sex, BMI primary diagnosis, causes of lumbar drainage, and complications of lumbar drainage, were retrieved. Drainage time was classified as <7 days and ≥7 days. CSF volume was divided into ≥200 ml/d and <200 ml/d. CSF properties were divided into normal and abnormal. Normal CSF was defined as CSF protein levels between 120–800 mg/L, colorless, and WBC of 0–50 × 10^6^/L. Abnormal CSF was defined as CSF protein ≥800 mg/L, turbid/bloody/yellow, and WBC >50 × 10^6^/L. The patient’s position after lumbar drainage is mainly prone or supine. Drainage tube choice manufacturer was either Medtronic or Branden.

### Statistical analysis

The categorical variables were expressed in numbers (percentages), and differences were evaluated using chi square tests or Fisher’s exact tests, the statistical significance level was set at *p* < 0.05. To detect risk factors associated with the incidence of lumbar drainage blockage, a univariate regression analysis was used. The risk factors with *p* < 0.05 in univariate logistic regression analysis were selected for further multiple regression analysis. The odds ratio (OR) and 95% confidence interval (CI) were calculated. All statistical analyses were performed using R software version 4.0 (R Core Team, R Statistical Computing Foundation, Vienna, Austria)^1^.

## Results

### Overview of lumbar drainage

This study analyzed the medical records of 637 patients who underwent lumbar drainage at our institution. Table 1 shows the clinical and demographic information of these patients. The main reasons for lumbar drainage included intracranial infection (48.2%, 307/637), intraventricular hemorrhage (23.4%, 149/637), subarachnoid hemorrhage (12.9%, 82/637), increased intracranial pressure (8.2%, 52/637) and CSF rhinorrhea/otorrhea (7.3%, 47/637). Complications of lumbar drainage included drainage tube blockage (13.7%, 87/637), CSF leakage (puncture point, 10.5%, 67/637), excessive drainage (2.7%, 17/637) and drainage tube prolapse (0.6%, 4/637).

**TABLE 1:** Demographic and clinical data.

Variable	Value (%)
No. of patients	637
**Sex**	
Female	329 (51.6%)
Male	308 (48.4%)
**Age**	
<60	277 (43.5%)
≥60	360 (56.5%)
**BMI**	
<24	230 (36.1%)
≥24	407 (63.9%)
**Primary diagnosis**	
Traumatic brain injury	187 (29.4%)
Vascular hemorrhage	213 (33.4%)
Intracranial tumor	219 (34.4%)
Brain abscess	18 (2.8%)
**Causes (LD)**	
Intracranial infection	307 (48.2%)
Intraventricular hemorrhage	149 (23.4%)
Subarachnoid hemorrhage	82 (12.9%)
Increased intracranial pressure	52 (8.2%)
CSF rhinorrhea/otorrhea	47 (7.3%)
**Complications (LD)**	175 (27.5%)
Drainage tube blockage	87 (13.7%)
CSF leakage (puncture point)	67 (10.5%)
Excessive drainage	17 (2.7%)
Drainage tube prolapse	4 (0.6%)

Among the 87 drainage tube blockage cases, drainage was achieved in 59 cases after recanalization by reducing the drainage height, adjusting the body position, repeatedly flushing with normal saline, achieving suction under negative pressure and pulling out part of the drainage tube, and the recanalization rate was 67.8% (59/87). Thirteen patients underwent lumbar cistern drainage again. Sixty-seven cases of cerebrospinal fluid leakage were combined with drainage tube blockage, which was pressurized and sutured through local wounds. After the drainage tube was opened, 54 cases had no cerebrospinal fluid leakage. The drainage tube was removed, and the wound was sutured in 13 cases. Excessive drainage (13 cases) is not the total amount of cerebrospinal fluid drained daily, but there is no guarantee of a continuous average drainage volume of cerebrospinal fluid per hour (average drainage speed <10–15 ml/h) (Nanidis et al., 2014). In some periods, the drainage speed of cerebrospinal fluid is too fast, which leads to intracranial hypotension symptoms (headache, occasional nausea, and vomiting) (Manley and Dillon, 2000; Açikbaş et al., 2002). After clamping the drainage tube, the patient improved after being placed in a supine position and infusion of normal saline. Drainage tube prolapse (4 cases) occurs when the drainage tube in the spinal canal is partially or completely pulled out of the body. After removal of the lumbar drainage tube, the patient was followed up for 1 week, and no symptoms related to the drainage tube were found.

### Predictors of lumbar drainage blockage

Lumbar drainage blockage was the most important complication of lumbar drainage (13.7%, 87/637), and possible risk factors for lumbar drainage blockage were analyzed.

Univariate analysis: The incidence of lumbar drainage obstruction was higher in patients with drainage duration ≥7 days than in patients with drainage duration <7 days (57.5 vs. 42.5%, *P* = 0.015, OR 1.79, 95% CI 1.14–2.85). CSF volume <200 mL/d was significantly more often associated with lumbar drainage blockage than CSF volume ≥200 mL/d (67.8 vs. 32.2%, *p* < 0.001, OR 5.20, 95% CI 3.22–8.57). Patients undergoing CSF leakage had lumbar drainage blockage significantly more often than those without CSF leakage (77.0 vs. 23.0%, *p* = 0.021, OR 1.90, 95% CI 1.14–3.31). Patients with abnormal CSF properties had lumbar drainage blockage significantly more often than those with normal CSF properties (88.5% vs. 11.5%, *p* = 0.014, OR 2.40, 95% CI 1.26–5.08) (Table 2).

**TABLE 2:** Baseline characteristics of the patients.

Variable	Overall	Non-blockage	Blockage	*P*
	(*n* = 637)	(*n* = 550)	(*n* = 87)	
Sex				0.306
Female	308 (48.4%)	261 (47.5%)	47 (54.0%)	
Male	329 (51.6%)	289 (52.5%)	40 (46.0%)	
Age (years)				0.876
<60	277 (43.5%)	238 (43.3%)	39 (44.8%)	
≥60	360 (56.5%)	312 (56.7%)	48 (55.2%)	
BMI				0.057
<24	230 (36.1%)	207 (37.6%)	23 (26.4%)	
≥24	407 (63.9%)	343 (62.4%)	64 (73.6%)	
Drainage time (day)				0.015
<7	351 (55.1%)	314 (57.1%)	37 (42.5%)	
≥7	286 (44.9%)	236 (42.9%)	50 (57.5%)	
CSF volume (mL/d)				<0.001
≥200	420 (65.9%)	392 (71.3%)	28 (32.2%)	
<200	217 (34.1%)	158 (28.7%)	59 (67.8%)	
CSF leakage (puncture point)				0.021
No	220 (34.5%)	200 (36.4%)	20 (23.0%)	
Yes	417 (65.5%)	350 (63.6%)	67 (77.0%)	
CSF properties				0.014
Normal	142 (22.3%)	132 (24.0%)	10 (11.5%)	
Abnormal	495 (77.7%)	418 (76.0%)	77 (88.5%)	
Causes				0.053
Others	179 (28.1%)	164 (29.8%)	15 (17.2%)	
Infection	306 (48.0%)	258 (46.9%)	48 (55.2%)	
Hemorrhage	152 (23.9%)	128 (23.3%)	24 (27.6%)	
Lesion location				0.208
Supratentorial	457 (71.7%)	400 (72.7%)	57 (65.5%)	
Subtentorial	180 (28.3%)	150 (27.3%)	30 (34.5%)	
Position				0.748
Supine	301 (47.3%)	258 (46.9%)	43 (49.4%)	
Lateral	336 (52.7%)	292 (53.1%)	44 (50.6%)	
Manufacturers				0.391
Medtronic	389 (61.1%)	340 (61.8%)	49 (56.3%)	
Branden	248 (38.9%)	210 (38.2%)	38 (43.7%)	

Multivariate analysis: we performed a multivariate logistic regression analysis to identify potential predictors of lumbar drainage blockage. Drainage time ≥7 days (*p* = 0.008, OR 1.92, 95% CI 1.18–3.11), CSF volume <200 mL/d (*p* < 0.001, OR 5.29, 95% CI 3.23–8.69), CSF leakage (*p* = 0.016, OR 1.96, 95% CI 1.12–3.42), and abnormal CSF properties (*p* = 0.017, OR 2.38, 95% CI 1.17–4.87) were identified as independent and significant predictors of lumbar drainage blockage (Table 3).

**TABLE 3:** Clinical risk factors for prediction of drainage tube blockage.

Variable	Univariable analysis		Multivariable analysis	
	OR (95% CI)	*P*	OR (95% CI)	*P*
Sex (female vs. male)	0.77 (0.49–1.21)	0.306		
Age (years) (<60 vs. ≥60)	0.94 (0.60–1.49)	0.876		
BMI (≥24 vs. <24)	1.67 (1.02–2.83)	0.057		
Drainage time (day) (<7vs. ≥7)	1.79 (1.14–2.85)	0.015	1.92 (1.18–3.11)	0.008
CSF volume (mL/d) (≥200 vs. <200)	5.20 (3.22–8.57)	<0.001	5.29 (3.23–8.69)	<0.001
CSF leakage (puncture point) (no vs. yes)	1.90 (1.14–3.31)	0.021	1.96 (1.12–3.42)	0.016
CSF properties (normal vs. abnormal)	2.40 (1.26–5.08)	0.014	2.38 (1.17–4.87)	0.017
Causes (others vs. infection)	2.02 (1.12–3.85)	0.053		
Causes (others vs. hemorrhage)	2.04 [1.03;4.14]	0.053		
Lesion location (supratentorial vs. subtentorial)	1.41 (0.86–2.26)	0.208		
Position (supine vs. lateral)	0.90 (0.57–1.43)	0.748		
Manufacturers (Medtronic vs. Branden)	1.26 (0.79–1.98)	0.391		

## Discussion

**FIGURE 1:**
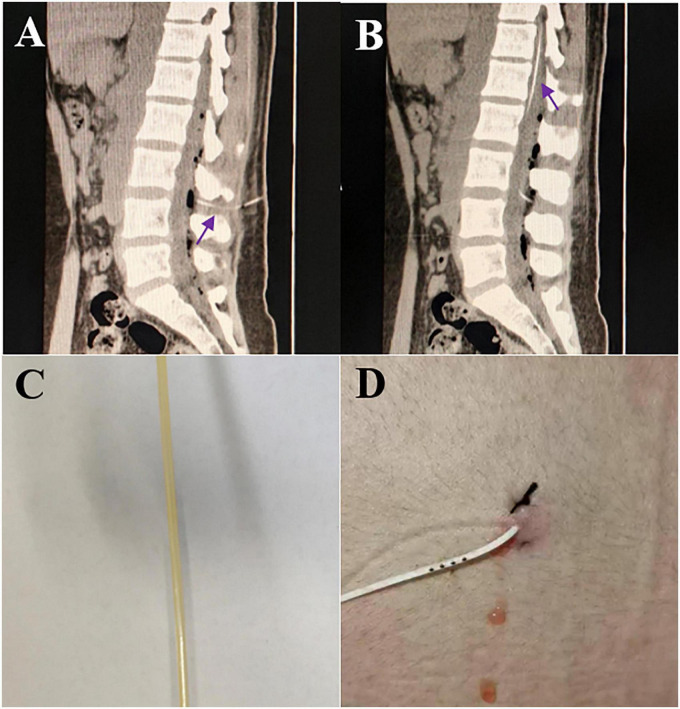
**(A)** The routine puncture site of lumbar cistern is between L3–4 (purple arrow); **(B)** the length of the drainage tube in the spinal canal is about 20 cm (purple arrow); **(C)** yellow, purulent cerebrospinal fluid flowing out of lumbar cistern canal; **(D)** intermittent bloody cerebrospinal fluid outflow at puncture site.

Continuous drainage of cerebrospinal fluid from lumbar cistern drainage is one of the most commonly used treatment techniques in neurosurgery (Figures 1A, B). Its main purpose is to drain bloody or contaminated cerebrospinal fluid outside the skull. It is also sometimes used to monitor and control intracranial pressure and promote wound healing of cerebrospinal fluid rhinorrhea/otorrhea. Drainage tube blockage is a common complication of continuous lumbar cistern drainage. Once the tube is blocked, the daily drainage volume of cerebrospinal fluid decreases significantly or is completely absent, which hinders the purpose of treatment. At the same time, the existence of the drainage tube increases the possibility of infection (Fried et al., 2016). We retrospectively analyzed the risk factors for lumbar cistern blockage. (1) Cerebrospinal fluid characteristics [infection (Figure 1C) or blood] are the main reason for lumbar cistern blockage. The protein content in the infected cerebrospinal fluid is increased, viscous infectious secretion can be seen in the cerebrospinal fluid with the naked eye, and the bloody cerebrospinal fluid is mixed with blood clots, a large amount of hemoglobin and inflammatory factors after red blood cell disintegration. These abnormal impurities are easily attached to the inner wall of the drainage tube. With the accumulation of impurities, the diameter of the drainage tube gradually narrows or is even blocked completely. (2) The leakage of cerebrospinal fluid at the lumbar cistern puncture point is an early sign of lumbar cistern blockage, which indirectly indicates that the lumbar cistern is blocked, leading to increased pressure in the spinal canal and forcing cerebrospinal fluid to flow out of the puncture point (Figure 1D). We compared BMI, and the thickness of subcutaneous fat at the puncture point prevented cerebrospinal fluid leakage to some extent, but there was no significant difference. The most fundamental reason is that the drainage tube is blocked, which leads to an increase in local cerebrospinal fluid accumulation in the spinal canal, and when the pressure reaches a certain point, the cerebrospinal fluid exudes from the puncture point. (3) Daily drainage volume: lumbar cistern drainage can achieve maximum drainage while ensuring safety. Studies have shown that continuous drainage of 200 ml every day is safe (Wang et al., 2013), and some studies have confirmed that the maximum drainage volume should not exceed 300 ml (Ma et al., 2021), and this needs to be comprehensively evaluated according to the characteristics of individual conditions and clinical manifestations. Ensuring continuous drainage can reduce the probability of drainage blockage by continuously flushing and pushing CSF, drainage wall-attached infectious substances, blood clots, or proteins out. (4) As the duration of drainage increased (≥7 days), the probability of drainage tube blockage increased significantly, and the probability of infection caused by catheterization also increased significantly.

The risk factors for drainage tube blockage are identified, and corresponding disposal measures should be taken according to the risk factors. In the case of infection or bloody cerebrospinal fluid, clogging is prevented by prophylactic repeated extrusion of the drainage tube or by injecting saline through a syringe and with intermittent suction. Ensuring continuous drainage of 200 ml of cerebrospinal fluid every day and a catheterization duration <7 days can effectively avoid the possibility of cerebrospinal fluid leakage and infection at the puncture point. If the tube is blocked for unknown reasons, and to ensure safety, one should reduce the height of the drainage tube (low intracranial pressure), adjust the body position (the punctured intervertebral space is forced to compress the drainage tube), pull out part of the drainage tube in the spinal canal (the side hole of the drainage tube is adsorbed to the arachnoid), and the drainage tube may recanalize.

Lumbar cisterna drainage is a routine operation in neurosurgery. At the same time, it is also a double-edged sword that can be used to shorten the recovery time of patients. If used improperly, it will cause many adverse consequences. Therefore, after catheterization of the lumbar cisterna, the daily drainage volume (200–300/ml/day) and retention time (<7 days) should be maintained in strict accordance with the guidelines or existing research conclusions. This should be performed with a relatively sterile operation and careful disposal to prevent lumbar cisterna blockage and ensure continuous drainage and achieve the purpose of disease treatment.

## Data availability statement

The raw data supporting the conclusions of this article will be made available by the authors, without undue reservation.

## Ethics statement

The studies involving human participants were reviewed and approved by Tangdu Hospital Ethics Committee. The patients/participants provided their written informed consent to participate in this study. Written informed consent was obtained from the individual(s) for the publication of any potentially identifiable images or data included in this article.

## Author contributions

MZ: conceptualization and writing—original draft. QT: methodology. XW, LL, and XD: writing—original draft. YQ: supervision. QC: writing—review and editing. All authors contributed to the article and approved the submitted version.

## Footnotes

1
http://www.R-project.org/
